# Supplementary material for: LDpred2: better, faster, stronger
Source: Bioinformatics. 2020 Dec 16;36(22-23):5424–31. doi: 10.1093/bioinformatics/btaa1029 (PMC8016455; doi:10.1093/bioinformatics/btaa1029)
Supplement: btaa1029_Supplementary_Data [file btaa1029_supplementary_data.pdf]

Supplementary Tables and Figures

Simulations

Method	all_40_300	all_40_3000	all_40_30000	all_40_300000	both_40	HLA_30_300	HLA_30_3000
LDpred1-inf	68.9 [68.4-69.3]	69.3 [68.9-69.6]	69.5 [69.2-69.7]	69.5 [69.3-69.6]	62.9 [61.6-64.3]	54.4 [52.9-55.9]	54.5 [53.7-55.5]
LDpred2-inf-perchr	70.3 [69.9-70.6]	70.0 [69.7-70.2]	69.9 [69.7-70.1]	69.9 [69.7-70.0]	74.2 [73.8-74.5]	75.6 [74.8-76.3]	75.7 [75.4-75.9]
LDpred1-grid	73.6 [72.0-75.4]	70.4 [69.8-70.9]	69.1 [68.8-69.3]	69.2 [69.0-69.4]	61.7 [60.4-63.1]	54.7 [53.9-55.5]	54.5 [53.8-55.1]
LDpred2-grid-nosp-perchr	81.7 [81.6-81.9]	78.9 [78.7-79.0]	70.6 [70.4-70.8]	69.3 [69.0-69.6]	72.8 [67.3-75.7]	76.4 [75.7-77.0]	76.4 [76.1-76.6]
LDpred2-grid-sp-perchr	81.7 [81.6-81.9]	78.8 [78.7-78.9]	70.5 [70.3-70.7]	69.4 [69.1-69.6]	72.8 [67.3-75.7]	76.5 [75.8-77.1]	76.5 [76.3-76.7]
LDpred2-auto-perchr	82.0 [81.8-82.2]	79.3 [79.2-79.4]	70.8 [70.6-71.0]	69.8 [69.6-70.0]	73.7 [73.3-74.2]	73.1 [72.0-74.1]	72.9 [71.6-74.0]

Table S1: Values from figure 1.

Method	10000	20000	50000	120000	300000
LDpred1-grid	56.1 [55.8-56.4]	59.3 [58.9-59.8]	66.3 [65.2-67.1]	70.7 [69.2-71.7]	70.4 [69.8-70.9]
LDpred1-inf	55.7 [55.4-56.0]	57.7 [57.4-58.0]	61.2 [60.9-61.5]	65.0 [64.7-65.3]	69.3 [69.0-69.7]
LDpred2-auto-gwide	55.9 [55.5-56.2]	59.3 [58.9-59.8]	67.5 [67.3-67.8]	74.7 [74.6-74.9]	79.3 [79.2-79.4]
LDpred2-auto-perchr	54.1 [53.7-54.5]	57.3 [56.8-57.8]	66.6 [66.3-66.9]	74.7 [74.5-74.8]	79.3 [79.2-79.4]
LDpred2-grid-nosp-gwide	55.7 [55.2-56.0]	59.6 [59.3-60.0]	67.4 [67.2-67.7]	74.6 [74.5-74.8]	79.1 [79.0-79.3]
LDpred2-grid-nosp-perchr	54.4 [54.0-54.8]	58.6 [58.1-59.1]	66.8 [66.5-67.1]	74.2 [73.9-74.5]	78.9 [78.7-79.0]
LDpred2-grid-sp-gwide	56.1 [55.7-56.5]	59.6 [59.3-60.0]	67.4 [67.2-67.7]	74.6 [74.4-74.8]	79.2 [79.1-79.3]
LDpred2-grid-sp-perchr	54.5 [54.2-54.9]	58.4 [57.9-58.9]	66.8 [66.6-67.1]	74.2 [73.9-74.4]	78.8 [78.7-78.9]
LDpred2-inf-gwide	55.5 [55.2-55.8]	57.7 [57.5-58.0]	61.4 [61.1-61.6]	65.4 [65.1-65.6]	69.9 [69.7-70.1]
LDpred2-inf-perchr	54.6 [54.3-55.0]	57.2 [56.8-57.5]	61.3 [61.0-61.5]	65.4 [65.2-65.6]	70.0 [69.7-70.2]

Table S2: Values from figures 2 and S2.

Real data

Method	Asthma	BRCA	CAD	MDD	PRCA	RA	T1D	T2D
LDpred2-grid-nosp-perchr	58.8 [58.5-59.1]	64.6 [64.1-65.1]	61.5 [61.0-62.0]	57.9 [57.5-58.2]	68.2 [67.6-68.7]	59.9 [59.2-60.6]	77.0 [75.2-78.8]	63.3 [62.9-63.7]
LDpred2-grid-sp-perchr	58.5 [58.2-58.8]	64.6 [64.1-65.0]	61.2 [60.8-61.7]	58.2 [57.8-58.6]	67.8 [67.3-68.4]	60.0 [59.3-60.7]	75.9 [74.0-77.7]	63.2 [62.7-63.6]
LDpred2-auto-perchr	58.2 [57.9-58.5]	65.6 [65.2-66.1]	62.1 [61.6-62.6]	58.9 [58.5-59.2]	70.2 [69.6-70.7]	59.7 [58.9-60.4]	76.6 [74.7-78.5]	63.9 [63.5-64.3]
LDpred2-inf-perchr	57.1 [56.8-57.4]	61.9 [61.4-62.4]	60.8 [60.4-61.3]	58.9 [58.5-59.2]	65.0 [64.4-65.6]	59.6 [58.9-60.3]	74.5 [72.6-76.3]	61.5 [61.1-61.9]
LDpred2-inf-gwide	57.2 [56.9-57.4]	61.6 [61.2-62.1]	61.6 [61.1-62.1]	59.0 [58.6-59.3]	64.4 [63.8-65.0]	59.3 [58.6-60.0]	71.0 [69.1-72.8]	61.6 [61.1-62.0]
LDpred2-grid-nosp-gwide	59.3 [59.0-59.6]	65.5 [65.0-66.0]	63.6 [63.2-64.1]	59.1 [58.7-59.4]	70.2 [69.6-70.7]	60.3 [59.6-61.0]	78.4 [76.7-80.2]	64.3 [63.8-64.7]
LDpred2-grid-sp-gwide	59.4 [59.1-59.7]	65.7 [65.3-66.2]	63.5 [63.1-64.0]	59.0 [58.7-59.4]	70.2 [69.6-70.7]	60.5 [59.8-61.3]	78.3 [76.5-80.0]	64.0 [63.6-64.5]
LDpred2-auto-gwide	58.4 [58.2-58.7]	65.6 [65.2-66.1]	61.8 [61.3-62.3]	59.0 [58.6-59.4]	70.1 [69.5-70.7]	59.7 [59.0-60.4]	77.7 [75.9-79.4]	63.8 [63.3-64.2]
LDpred1-grid	58.6 [58.3-58.8]	58.9 [58.4-59.4]	60.3 [59.9-60.8]	56.4 [56.1-56.8]	63.5 [62.9-64.1]	59.1 [58.4-59.8]	57.4 [55.5-59.2]	50.3 [49.8-50.7]
LDpred1-inf	56.9 [56.6-57.1]	58.8 [58.3-59.3]	59.3 [58.9-59.8]	56.5 [56.1-56.8]	61.6 [61.0-62.3]	59.5 [58.8-60.2]	57.7 [55.8-59.5]	51.8 [51.4-52.2]

Table S3: Values from figures 3 and S3.

Method	Asthma	BRCA	CAD	MDD	PRCA	RA	T1D	T2D
LDpred2-inf-gwide	57.2 [56.9-57.4]	61.6 [61.1-62.1]	61.6 [61.1-62.0]	59.0 [58.6-59.3]	64.4 [63.8-65.0]	59.3 [58.6-60.0]	71.0 [69.1-72.9]	61.6 [61.1-62.0]
LDpred2-grid-nosp-gwide	59.3 [59.0-59.6]	65.5 [65.0-66.0]	63.6 [63.2-64.1]	59.1 [58.7-59.4]	70.2 [69.6-70.8]	60.3 [59.6-61.0]	78.4 [76.7-80.1]	64.3 [63.9-64.7]
LDpred2-grid-sp-gwide	59.4 [59.1-59.7]	65.7 [65.3-66.2]	63.5 [63.1-64.0]	59.0 [58.7-59.4]	70.2 [69.6-70.7]	60.5 [59.8-61.3]	78.2 [76.5-80.0]	64.0 [63.6-64.5]
LDpred2-auto-gwide	58.4 [58.2-58.7]	65.6 [65.2-66.1]	61.8 [61.3-62.3]	59.0 [58.6-59.4]	70.1 [69.5-70.7]	59.7 [59.0-60.5]	77.7 [75.9-79.4]	63.8 [63.3-64.2]
SCT	58.3 [58.0-58.6]	62.3 [61.8-62.8]	60.6 [60.1-61.1]	57.4 [57.1-57.8]	66.5 [65.9-67.1]	57.4 [56.7-58.1]	72.4 [70.5-74.2]	62.5 [62.0-62.9]
C+T	56.7 [56.4-57.0]	62.9 [62.5-63.4]	61.6 [61.1-62.1]	58.5 [58.1-58.9]	67.3 [66.7-67.9]	59.1 [58.4-59.8]	74.4 [72.6-76.2]	59.9 [59.4-60.3]
lassosum	57.6 [57.3-57.8]	65.2 [64.7-65.6]	62.5 [62.1-63.0]	59.1 [58.8-59.5]	69.3 [68.7-69.9]	59.3 [58.5-59.9]	74.3 [72.5-76.1]	62.7 [62.3-63.2]
lassosum-auto	57.6 [57.3-57.8]	65.1 [64.7-65.6]	61.4 [61.0-61.9]	54.9 [54.6-55.3]	69.3 [68.7-69.8]	58.2 [57.5-58.9]	75.8 [74.1-77.5]	62.4 [62.0-62.8]
PRS-CS	57.1 [56.8-57.4]	63.3 [62.8-63.7]	61.8 [61.4-62.3]	57.5 [57.2-57.9]	67.5 [66.9-68.1]	59.2 [58.5-59.9]	74.2 [72.4-76.0]	62.2 [61.7-62.6]
PRS-CS-auto	56.6 [56.3-56.8]	63.4 [62.9-63.8]	60.9 [60.4-61.3]	53.9 [53.5-54.3]	67.2 [66.6-67.7]	58.6 [57.9-59.3]	73.9 [72.1-75.7]	62.4 [62.0-62.9]
SBayesR	57.6 [57.4-57.9]	65.7 [65.2-66.2]	62.2 [61.7-62.6]	58.8 [58.5-59.2]	69.6 [69.1-70.2]	56.2 [55.5-56.9]	58.1 [56.1-60.2]	64.1 [63.7-64.5]

Table S4: Values from figures 4 and S1.

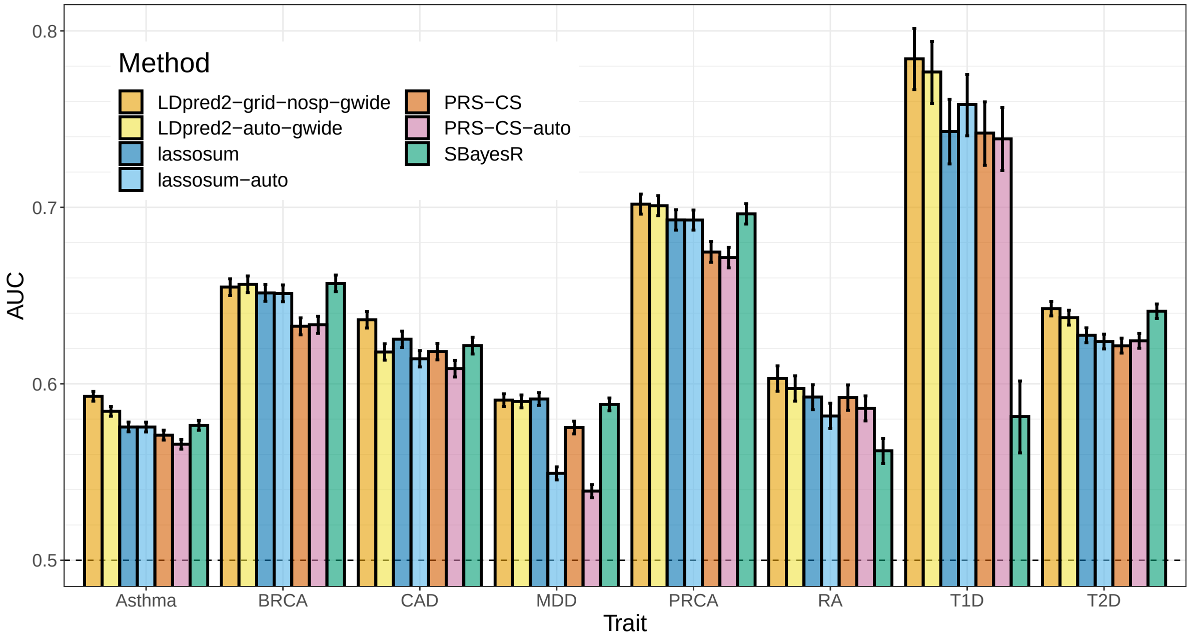

Figure S1: Both “grid” and “auto” variants are compared in real data applications for LDpred2, lassosum, PRS-CS and SBayesR. Note that SBayesR is such an “auto” model, but does not have any “grid” counterpart. Bars present AUC values on the test set of UKBB (mean and 95% CI from 10,000 bootstrap samples). See corresponding values in table S4.

Running LDpred2 per chromosome or genome-wide?

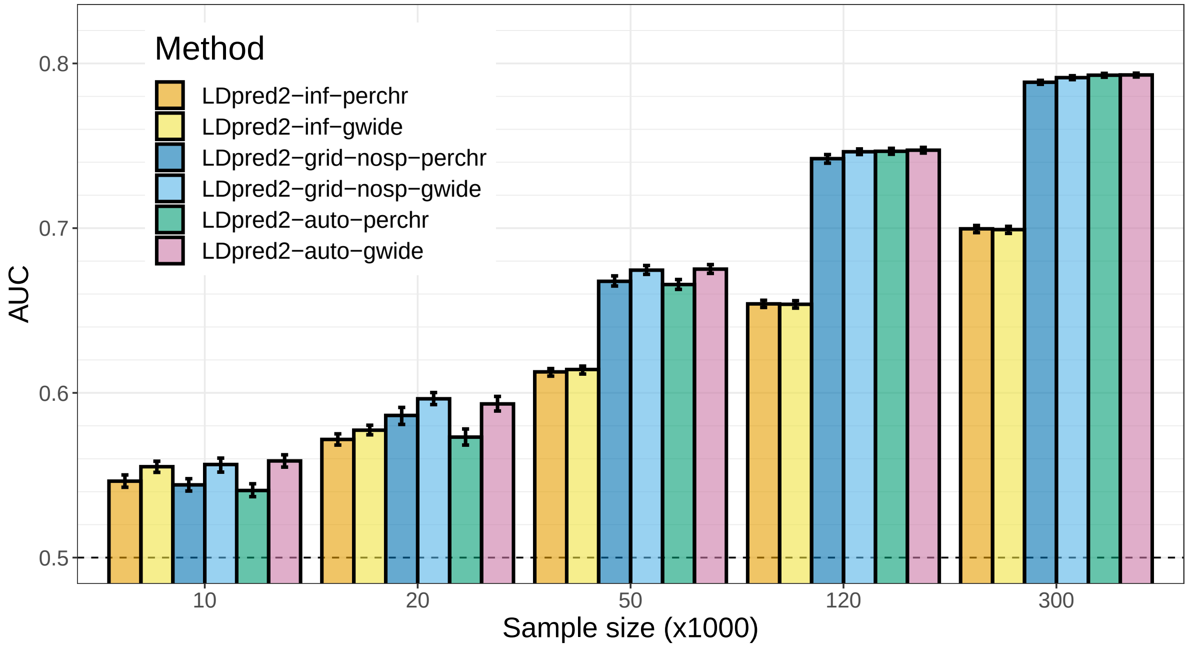

Figure S2: LDpred2 models, run either per chromosome or genome-wide, are compared when varying GWAS sample size in scenario “all_40_3000”. Bars present the mean and 95% CI of 10,000 non-parametric bootstrap replicates of the mean AUC of 10 simulations for each scenario. See corresponding values in table S2.

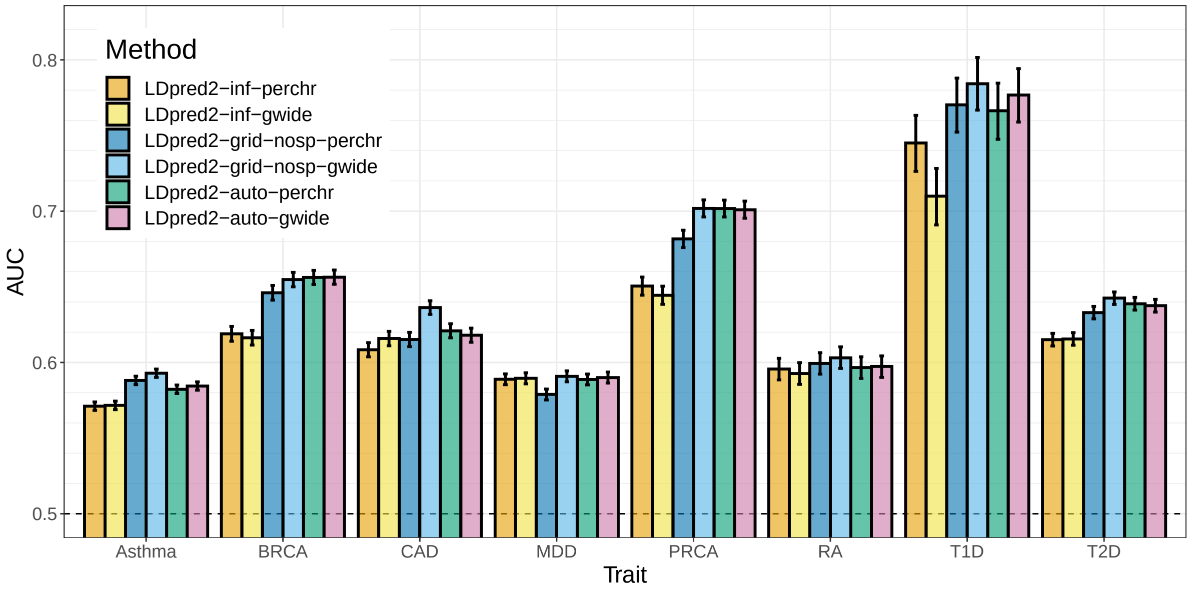

Figure S3: LDpred2 models, run either per chromosome or genome-wide, are compared in real data applications. Bars present AUC values on the test set of UKBB (mean and 95% CI from 10,000 bootstrap samples). See corresponding values in table S3.

Estimation of parameters in LDpred2

Trait	h2_ldsc_obs	sparsity_grid	p_auto	h2_auto_obs	prev_pop	h2_auto_liab
Asthma	0.104	0.544	0.000738	0.0534	0.15	0.064
BRCA	0.127	0.414	0.00474	0.141	0.0758	0.136
CAD	0.0819	0.441	0.000978	0.0428	0.0619	0.0387
MDD	0.0923	0.377	0.0675	0.0877	0.0893	0.089
PRCA	0.188	0.516	0.00181	0.189	0.0546	0.164
RA	0.416	0.193	0.0774	0.562	0.0286	0.406
T1D	0.955	0.498	0.00482	1.4	0.00244	0.575
T2D	0.148	0.498	0.0031	0.131	0.0512	0.112

Table S5: Parameters obtained when running LDpred2 genome-wide for all 8 real traits analyzed here. h2_ldsc_obs: SNP heritability estimate (on the observed scale) from LD score regression (with intercept); sparsity_grid: sparsity of resulting effects from LDpred2-grid-sp; p_auto: estimate from LDpred2-auto of the proportion of causal variants; h2_auto_obs: SNP heritability estimate from LDpred2-auto; prev_pop: assumed population prevalence based on prevalence in the UK Biobank; h2_auto_liab: transformation of h2_auto_obs on the liability scale when assuming population prevalence prev_pop.

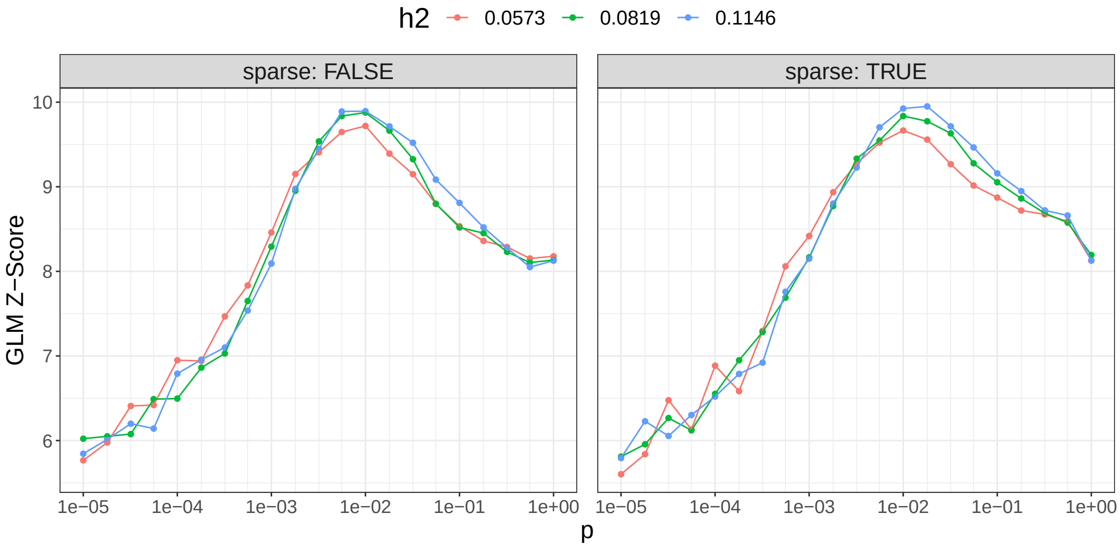

Figure S4: Results for all 126 parameter combinations of LDpred2-grid run genome-wide when applied to summary statistics for CAD.

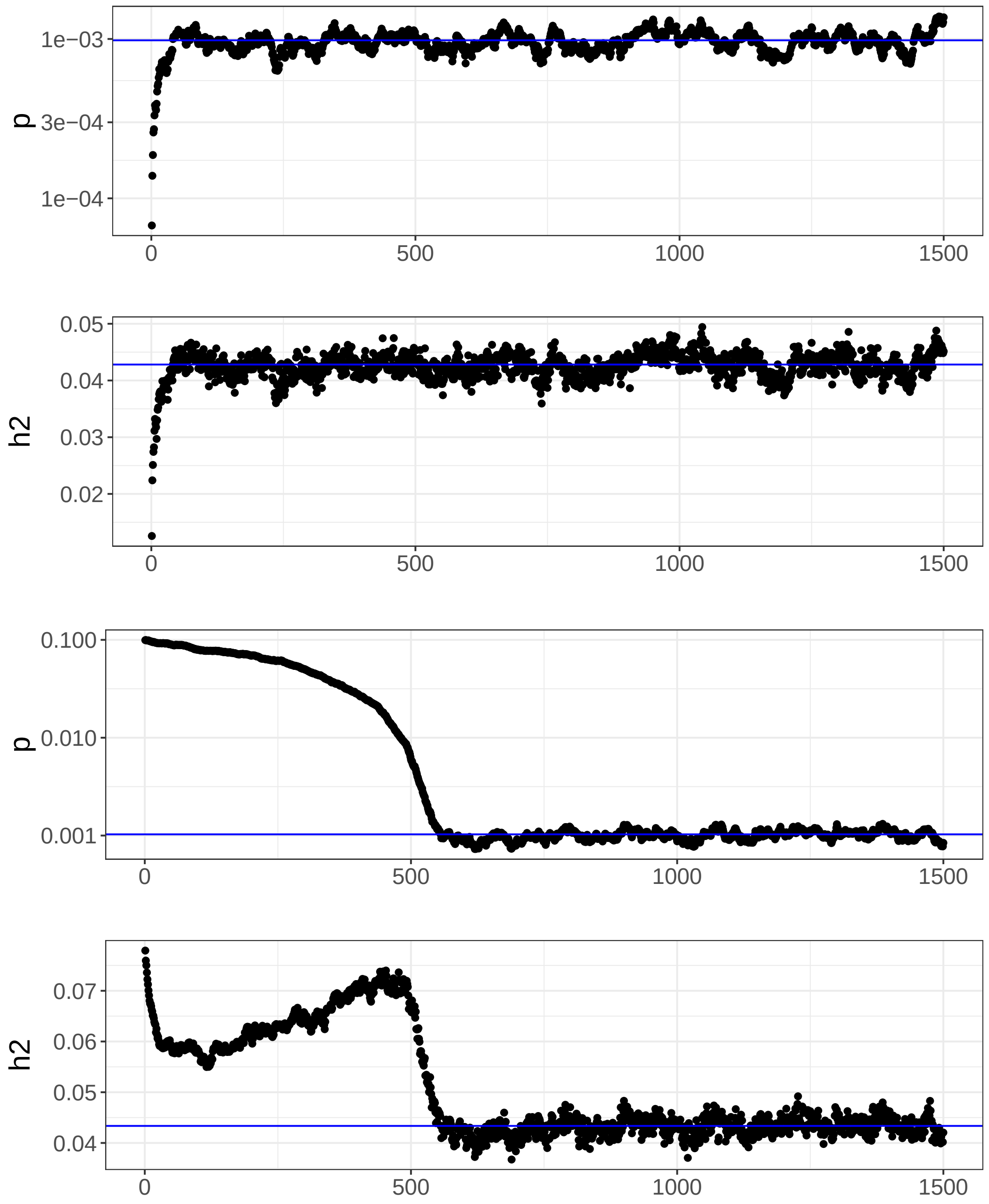

Figure S5: Paths of LDpred2-auto-gwide parameters, the SNP heritability h^2 and the proportion of causal variants p, for CAD. We show these for two different starting values for p, with an initial p of 0.0001 at the top and initial p of 0.1 at the bottom.

Quality control of summary statistics

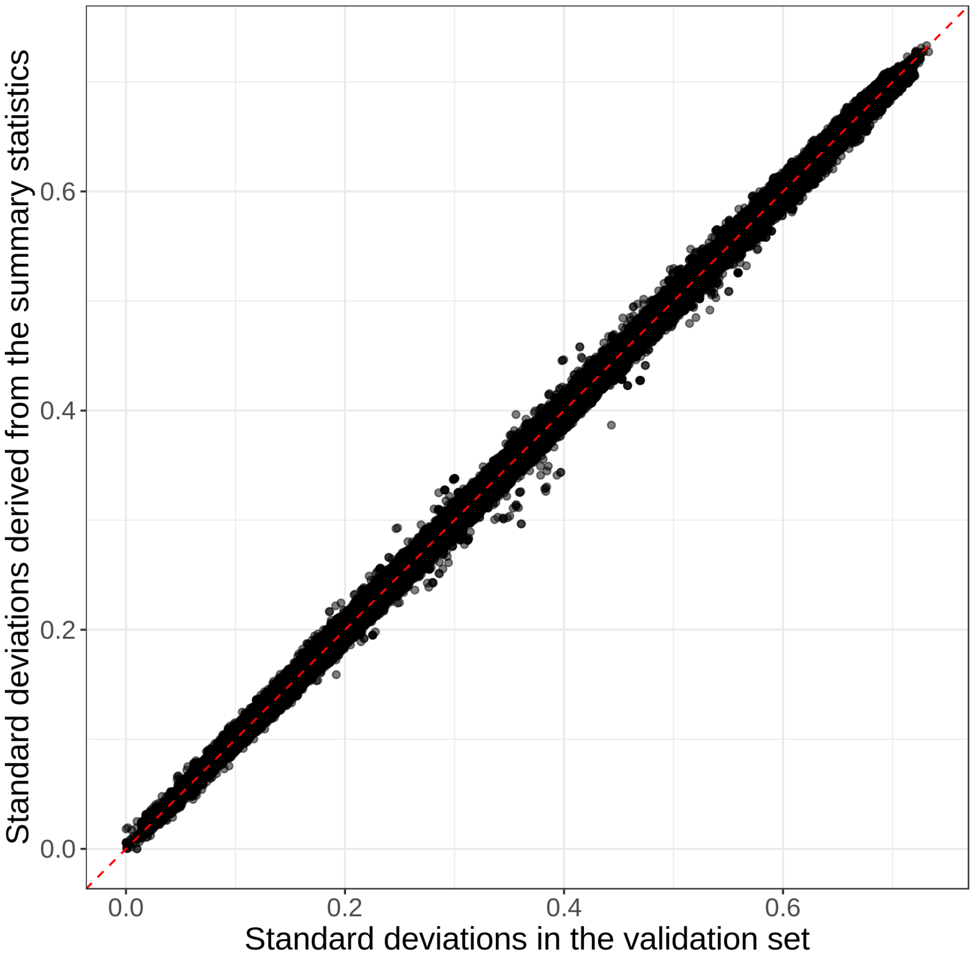

Figure S6: In simulations, standard deviations derived from summary statistics based on equation (3) versus the standard deviations of genotypes of individuals in the validation set.

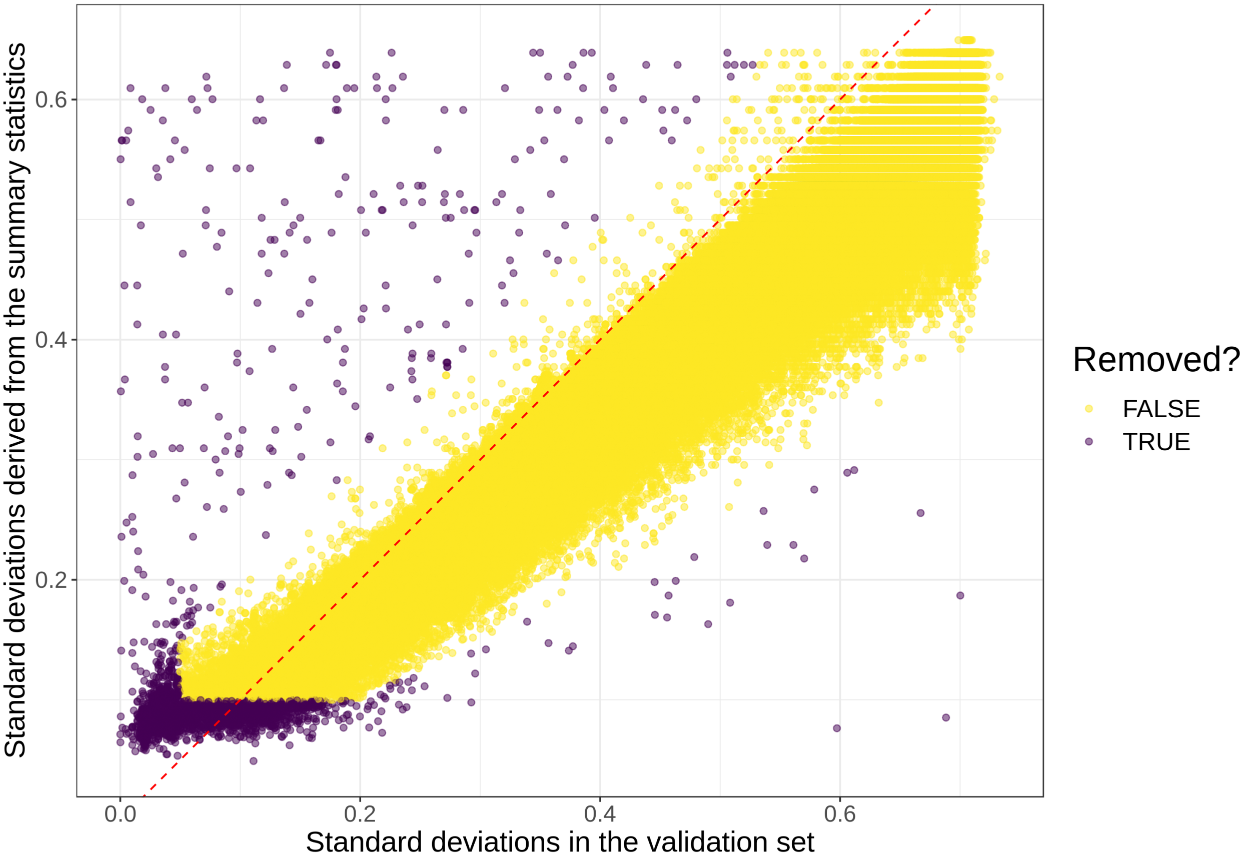

Figure S7: Standard deviations derived from summary statistics of breast cancer (BRCA) based on equation (3) versus the standard deviations of genotypes of individuals in the validation set. Coloring shows the quality control applied in this paper.

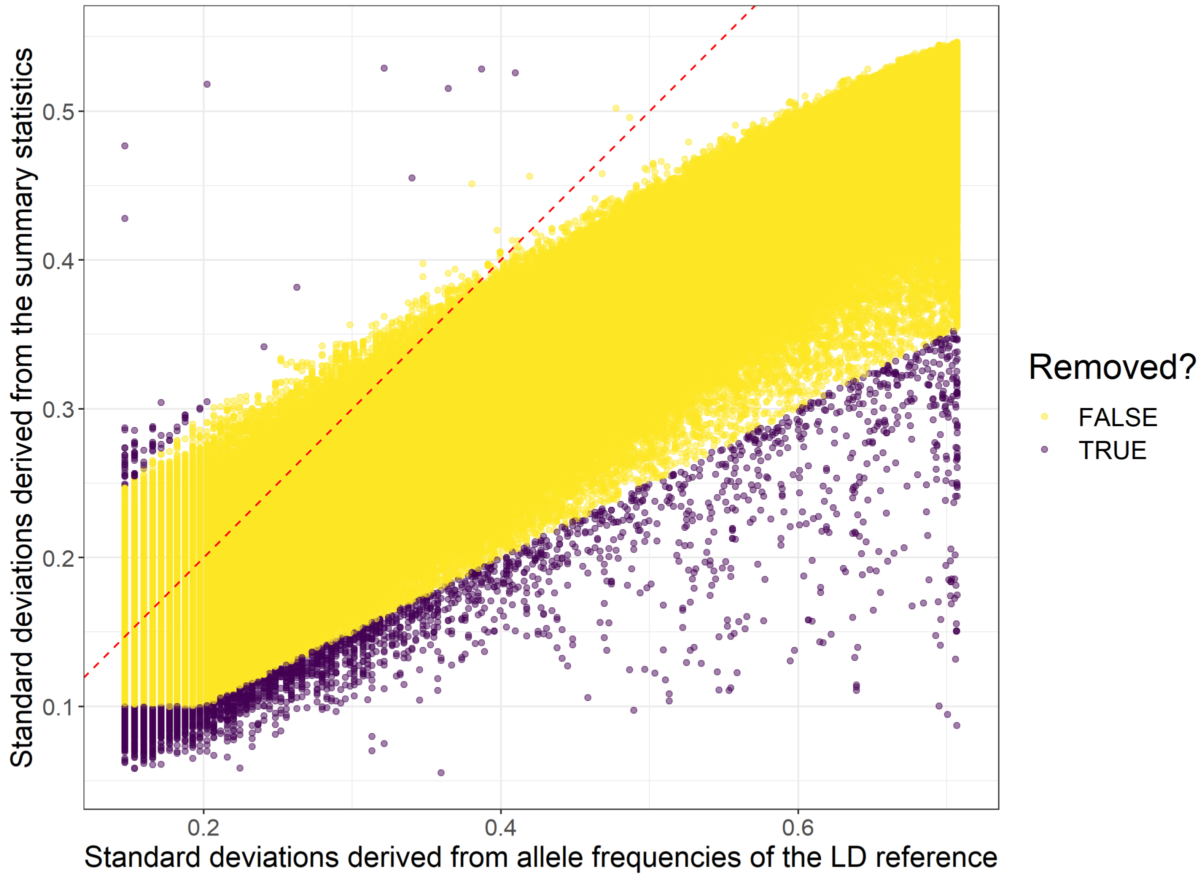

Figure S8: Standard deviations derived from summary statistics of coronary artery disease (CAD) based on equation (3) versus the standard deviations of genotypes of individuals in the validation set. Coloring shows the quality control applied in this paper.

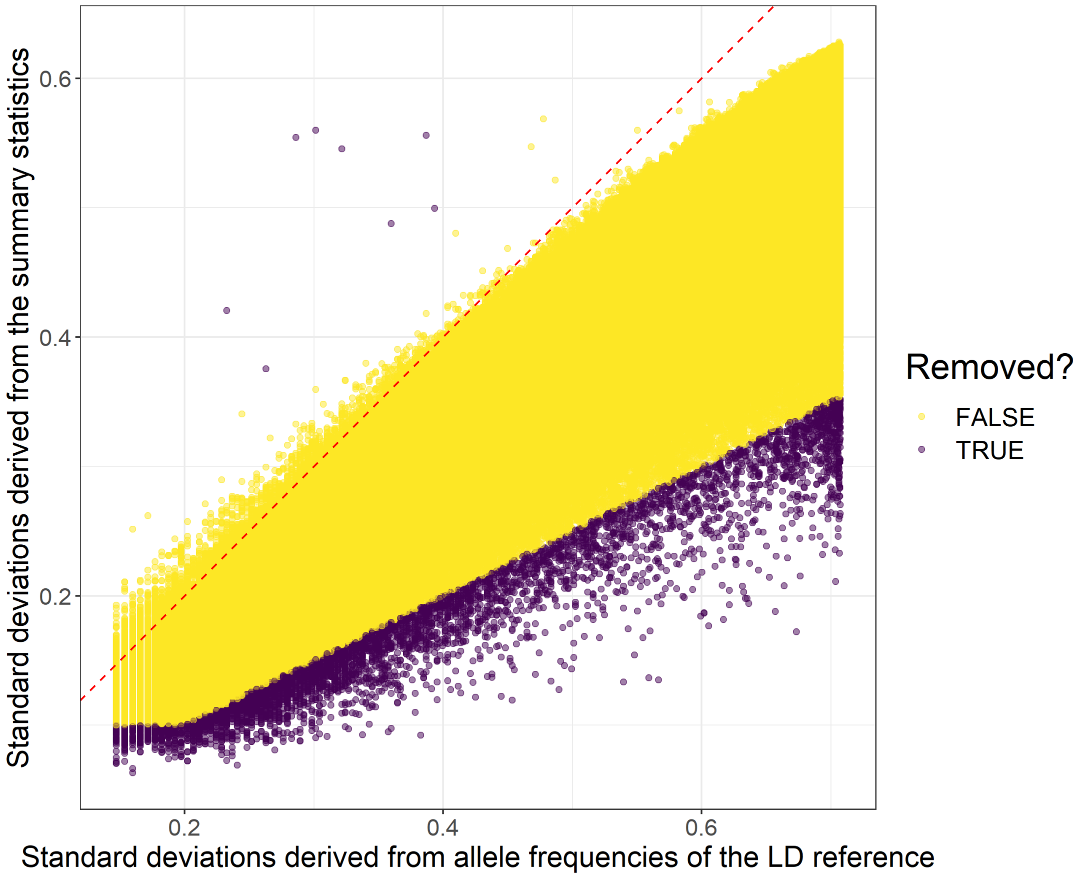

Figure S9: Standard deviations derived from summary statistics of asthma based on equation (3) versus the standard deviations of genotypes of individuals in the validation set. Coloring shows the quality control applied in this paper.

Providing an LD reference

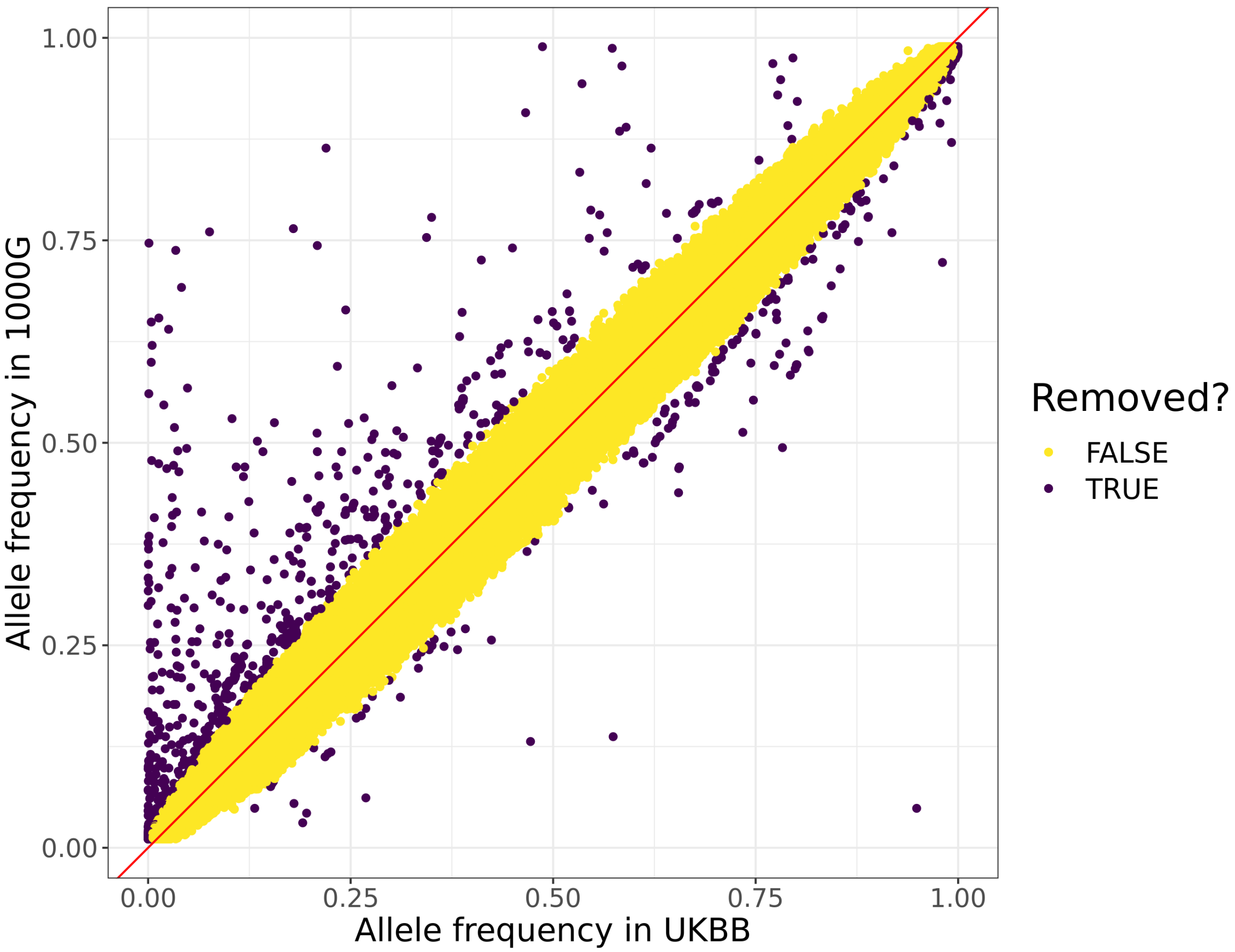

Figure S10: Allele frequencies for HapMap3 variants in the 1000 Genomes and in UK Biobank. Variants are removed when the difference between these two frequencies is very significant ($p < 10^{-5}$). Variants kept are used to provide an LD reference based on the UK Biobank data.
